# TILs and Anti-PD1 Therapy: An Alternative Combination Therapy for PDL1 Negative Metastatic Cervical Cancer

**DOI:** 10.1155/2020/8345235

**Published:** 2020-09-07

**Authors:** Huanhuan Yin, Wei Guo, Xiangling Sun, Ruili Li, Cuihua Feng, Yujie Tan

**Affiliations:** Department of Obstetrics, Luoyang Central Hospital Affiliated to Zhengzhou University, Luoyang 471009, China

## Abstract

**Background:**

We investigated the efficacy of TILs and anti-PD1 combination therapy in patients with metastatic cervical cancer with low MSI expression and PDL1-negative.

**Methods:**

A total of 80 patients were put on TILs and anti-PD1 combination therapy, and the progression-free survival time (PFS) and overall survival time (OS) were assessed by Kaplan–Meier analysis. Univariate and multivariate analyses were performed to identify factors that could predict the prognosis of metastatic cervical cancer in the previously described patients.

**Results:**

The objective response rate was 25%, whereas the mPFS and mOS were 6.1 and 11.3 months, respectively. The therapeutic efficacy was influenced by the characteristics of TILs, infection with HPV, and development of fever just after the therapy.

**Conclusion:**

Overall, our results show that the combination therapy of TILs and anti-PD1 significantly improves the prognosis of metastatic cervical cancer.

## 1. Introduction

Globally, cervical cancer is not only one of the major health complications affecting women but also the fourth most prevalent cancer in women [1].Epidemiologically, 85% of all cancer cases occur in developing countries, where cervical cancer leads in the number of cancer-related mortality among women [2]. In 2019 alone, 13170 new cases of uterine cervical carcinomas were reported in the united states, where 4250 people died of the disease [3]. Although the prevalence of cervical cancer is generally decreasing among women in the United States, the incidence rates remain high among Hispanic/Latino, Black, and Asian women [4–7]. The primary treatment for early-stage cervical cancer is either surgery or radiation therapy. On a positive note, the 5-year progression-free survival rate (PFS) of patients with cervical cancer exceeds 70% [8]. However, metastatic types or recurrent lesions are not amenable to radical local excision or regional radiation and generally have a poor prognosis. Consequently, they are treated with the more toxic palliative platinum-based chemotherapy. Median overall survival (OS) rates range from 6.5 to 13.3 months for chemotherapy regimens alone, and 6.5 to 16.8 months for bevacizumab-containing regimens [9–12]. Unfortunately, there are few effective therapeutic options for patients with recurrent tumors which are resistant to first-line chemotherapy, and thus, this group of patients exhibit poor prognosis to the available regimens. Therefore, metastatic cervical cancer presents one of the biggest challenges in clinical practice. In view of this, there is an urgent need to develop new therapeutic options, effective against metastatic cervical cancer.

Under normal physiological conditions, immune checkpoints play a crucial role in the prevention of autoimmunity [13]. To survive in the body, cancer cells express programmed death-ligand 1 (PD-L1), a molecule that can modulate the immune checkpoints and thus downregulate the generation of tumor-specific T cells. In effect, cancer cells protect themselves against immune attack [14]. Immune checkpoint inhibitors reduce the interaction between cancer cells and activated T cells which destroy malignant targets. Immunotherapy with immune checkpoint inhibitors has emerged as a novel treatment for patients with advanced cancers. Recent evidence has demonstrated the potential efficacy of immune checkpoint inhibitors in cervical cancer [15]. The objective response rate (ORR) for these treatments in recurrent and/or advanced cervical cancer ranges from 12.2 to 26% [16]. However, the selection of patients that potentially responds positively to this therapy remains challenging. Increased expression of PD-L1 on tumor and immune cells and high levels of microsatellite instability (MSI-high) have been associated with better response to immunotherapies [17]. However, the response rate of metastatic cervical cancer in patients with PDL1-negative and low MSI expression is relatively poor when put on anti-PD1 therapy. Therefore, anti-PD1 therapy alone may not be an effective treatment method for metastatic cervical cancers, particularly in patients testing negative for PDL1 and low MSI expression.

Adoptive T cell therapy (ACT) is an emerging cancer treatment modality that involves systemic infusion of therapeutic T cells. It has been shown to induce complete tumor responses in some patients with B cell malignancies or metastatic melanoma [18]. In one phase II study on tumor-infiltrating lymphocyte (TIL) therapy for the treatment of patients with metastatic carcinomas associated with human papillomavirus (HPV), it was found that TILs yielded an objective response rate (ORR) of 28% (5/18) in patients with cervical cancer [19]. Interestingly, the reactivity of HPV to the infused T cells together with repopulation of peripheral blood with HPV-reactive T cells positively correlated with better clinical response [20]. These findings support the concept that TILs therapy can inhibit metastatic cervical cancer. The effects of anti-PD1 therapy are mediated by TILs in the tumor microenvironment; therefore, a combination of anti-PD1 therapy and TILs may confer superior antitumor effects against metastatic cervical cancer than current therapies.

This study was primarily aimed at assessing the response to a combination of adoptive TILs and anti-PD1 in patients with chemotherapy-resistant cervical cancer. We also sought to determine potential biomarkers associated with better response to TILs and anti-PD1 combination therapy. This will help in identifying patients most likely to respond better to the newly proposed therapy.

## 2. Materials and Methods

### 2.1. Study Design

This single-center clinical study was approved by the Ethics Committee at the Affiliated Luoyang Central Hospital of Zhengzhou University. All methods and procedures associated with this study were conducted in accordance with the Good Clinical Practice guidelines and accorded ethically with the principles of the Declaration of Helsinki and local laws. All authors had access to the study data and reviewed and approved the final manuscript. Infusions of anti-PD1 therapy (nivolumab, 3 mg/kg) were administered to the patients at our department two weeks for one cycle. All patients received at least 8 cycles of infusions or received cycles until they experienced disease progression or unacceptable adverse effects (AEs) or withdrew this study. At the first cycles of anti-PD1 therapy, TILs were transfused into patients. Patients with disease progression received multidisciplinary synthetic therapy and best support cares. After treatment, all the patients received follow-up to examine the tumor status every 3 months. The follow-up deadline was June 20, 2020.

### 2.2. Patients Selection

Eighty patients with a clinical diagnosis of cervical cancer were enrolled in this study. In addition, the study group had experienced disease progression after first-line chemotherapy. Other inclusion criteria included the following: (1) discontinuing any cancer therapy before enrollment, (2) be an aged above 35 years old, (3) life expectancy of more than 3 months, (4) Eastern Cooperative Oncology Group (ECOG) performance status of 0-1, (5) adequate organ function, and (6) lesions that can be assessed using the standard response evaluation criteria in solid tumors (RECIST 1.0 version 1.1) guidelines [21]. The following exclusion criteria were applied: previous treatment with anti-CTLA4 or anti-PD1/PDL1 therapy, any form of primary immunodeficiency or history of autoimmune diseases, ongoing systemic infections, and concurrent systemic steroid therapy, and recruitment into other clinical trials. All participating patients provided informed consent.

### 2.3. Outcome Measures

The primary endpoint for this research was to evaluate the safety and AEs associated with a combination therapy of TILs and nivolumab in patients with cervical cancer. Secondary endpoints included the assessments of the objective response rate (ORR), progression-free survival time (PFS), and overall survival time (OS). Safety evaluations primarily consisted of any abnormalities identified clinically or in the laboratory, arising at any stage of the study up until two weeks after the last infusion of nivolumab. AEs were evaluated based on the guidelines set by the National Cancer Institute (NCI) on Common Toxicity Criteria (CTC), version 4.0 [22]. Therapy-associated AEs were assessed during the treatment and observation periods, where the most severe events for each patient based on the grading scores were also recorded. The cancer lesions were evaluated using computed tomography (CT) or magnetic resonance imaging (MRI) after every 3 months. The ORR was assessed using RECIST version 1.1 [21]. The ORR was defined as complete response (CR) plus partial response (PR), and disease control rate (DCR) was defined as CR plus PR plus stable disease (SD). Potential prognostic factors were also evaluated using various univariate and multivariate analyses. The PFS was calculated from the date a patient was put on the immunotherapy to the time the disease worsens. Patients who did not show any improvement were censored at the time of last contact. The OS was calculated from the date the first immunotherapy was administered to the time of death, and patients who were alive at the time of last contact were also censored.

### 2.4. Generation of TILs

TILs were obtained by culturing fresh tumor tissues from metastatic sites, extracted from each patient using a thick needle puncture. The tumor tissues were evaluated by two independent pathologists before the cultures were made. The experiment was performed as previously described [19, 23, 24]. Briefly, tumor tissues were sliced into small pieces of about 2 to 3 mm^3^ using a scalpel. Cell suspensions were made by digesting the tissues using collagenase type IV (Sigma-Aldrich, St. Louis, MO, USA, 1 mg/mL), DNase I (Sigma-Aldrich, St. Louis, MO, USA, 2 U/mL), and hyaluronidase type V (Sigma-Aldrich, St. Louis, MO, USA, 0.5 U/mL) for approximately 3 hours at room temperature. The single-cell suspensions were filtered, washed twice with phosphate-buffered saline (PBS), and incubated in a 12-well plate at a concentration of 1.0 × 10^6^ TILs/mL in an X-VIVO medium (Muenchensteinerstrasse 38 CH-4002 Basel, Switzerland) containing 7000 IU/mL of recombinant human interleukin-2 (rhIL-2, Novartis, UK). The day of culturing was considered as day 0. The cell suspensions were removed and further purified using Ficoll gradient at day 1. The purified bulk of TIL were recultured in X-VIVO medium mixed with 7000 IU/mL rhIL-2 and maintained at a concentration of 1 − 2 × 10^6^ cells/mL until all other cells (including cancer cells) were eliminated. This was done to achieve a concentration of at least 5 × 10^7^ TIL cells/ml. In general, the culturing process was performed for approximately 10 to 14 days. The cultured TIL cells were immediately incubated with anti-CD3 antibody (GE Healthcare Biosciences, Pittsburgh, PA, USA; 30 ng/mL) mixed with 1000 IU/mL rhIL-2 for large-scale production of TIL. In the end, about 5 × 10^9^ TIL cells were harvested, purified, immunophenotyped, and infused back into patients.

### 2.5. TILs Immunophenotyping

Immunophenotyping of TILs was performed as previously described [23, 24]. The postculture phenotypes of TILs were characterized using flow cytometry. The cells were first labeled using anti-CD3-FITC (Cat#: 555339, 1.5 *μ*l/10^6^ cells), anti-CD4-APC-Cy7 (Cat#: 557871, 2 *μ*l/10^6^ cells), anti-CD8-BV786 (Cat#: 563823, 2 *μ*l/10^6^ cells), anti-CD56-BV421 (Cat#: 562752, 3 *μ*l/10^6^ cells), and anti-PD1-PE-Cy7 (Cat#: 561272, 5 *μ*l/10^6^ cells) after a 30 minutes culture on ice and darkness. Thereafter, the cells were washed once with PBS and resuspended in 400 *μ*l PBS. Live and dead cells were distinguished using 7ADD. They were then run on a BD Fortessa (BD Bioscience). Negative control of each channels was labeled using Fluorescence minus one (FMO) technique as previously described [25]. The data generated in the flow cytometry was analyzed using the FlowJo software. To analyze T regulatory cells, FoxP3 staining was performed using an intracellular staining protocol following manufacturers' protocol. Next, anti-CD3 and anti-CD4 were first stained for 30 minutes on ice in the dark. TILs were washed, fixed with the labeled anti-CD3 and anti-CD4, and permeabilized using BD Fix Buffer I (Cat#: 557870, BD bioscience, USA) and Perm Buffer III (Cat#: 558050, BD bioscience, USA) following the manufacturers' protocol. The cells were washed thrice with Perm Buffer III and incubated for 30 minutes with anti-FoxP3-V450 (Cat#: 560460, 5 *μ*l/10^6^ cells) under ice and in darkness. The cells were then run on a BD Fortessa (BD Bioscience). Fluorescence minus one (FMO) staining was used to generate negative controls. The data generated by flow cytometry was analyzed using the Flowjo software. The gating strategies for T cell phenotype were as follows: (1) plot of FSC-A and SSC-A; (2) plot of FSC-A and FSC-H to gate single cells; (3) plot of 7AAD negative populations to gate live cells. Based on these gating, we then gated CD3^+^ T cell populations. For CD3^+^CD4^+^ T cells or CD3^+^CD8^+^ T cells or CD3^+^CD56^+^ NKT cells, we gated CD4^+^ or CD8^+^ or CD56^+^ populations followed by CD3^+^ populations. For NK cells populations, we gated CD3^−^ populations and then gated CD56^+^ populations. To determine the percentage of PD1^+^ cells on CD3^+^CD8^+^ T cells, we firstly gated CD3^+^ T cells, then gated CD8^+^ T cells followed by CD3^+^ populations. Finally, we gated PD1^+^ populations followed by CD3^+^CD8^+^ populations. For Treg cells population, we first gated CD3^+^CD4^+^ T cells population and then gated FoxP3^+^ population.

### 2.6. Cytotoxicity and Cytokines Production of TILs

Antitumor activity of TILs was assessed using an overnight cytotoxicity assay. Here, Hela cell line was used as target. Cytotoxic activity was determined using a lactate dehydrogenase (LDH) release assay as previously described. This nonradioactive assay is a colorimetric alternative to the ^51^Cr release assay that quantitatively measures LDH released upon cell lysis. In each assay, effector cell concentration at 40 : 1, 20 : 1, and 10 : 1 was evaluated. For the cytokines production of TILs, we measured the release of IFN-*γ* (BV650-conjugated, Cat#: 563416), IL-2 (BV510-conjugated, Cat#: 564167), and TNF-*α* (APC-conjugated, Cat#: 551384), using an intracellular staining protocol following manufacturers' protocol for both fresh TILs and cultured TILs. We compared the expression of IFN-*γ*, IL-2, and TNF-*α* on CD3^+^ T cells before and after culture. The intracellular staining method is the same to FoxP3 staining.

### 2.7. Statistical Analysis

GraphPad Prism 7.0 and Spss17.0 software was used for statistical analysis. PFS and OS were calculated by Kaplan–Meier. OS and PFS were calculated from the start of TILs therapy. Univariable and multivariable Cox proportional hazards regression models were used to estimate hazard ratios along with associated confidence intervals and *P* values. Other data used *t*-test or *χ*^2^-test. For all statistical analyses, significance is indicated as at least *P* < 0.05.

## 3. Results

### 3.1. Patient Characteristics

Between June 2017 and March 2019, 80 patients with persistent metastatic cervical cancer after developing resistance to first-line chemotherapy were enrolled and put on a combination therapy of TILs and nivolumab. Briefly, 25% of the patients received a combination of first-line chemotherapy and bevacizumab, whereas the rest, 75% were put on chemotherapy alone. The median age of patients was 45, with ages ranging from 35 to 65 years. The most common sites for metastatic cervical cancer were lungs and lymph nodes, which accounted for 55% and 40%, respectively. HPV subtyping for strain 6, 11, 16, 18, or 33 was also performed for patients who tested positive for the virus. Notably, all the patients were PD-L1-negative (both tumor cells and immune cells were negative) and exhibited low expression of MSI. Detailed characteristics of the patients are shown in Table 1.

### 3.2. Phenotype for TILs

Each patient was infused with an average of 50 × 10^9^ TILs (range, 35 − 88 × 10^9^). The phenotype of TILs was examined by flow cytometry and representative dot plots for T cell phenotypes are shown in supplementary Figure 1. The TILs primarily comprised of CD3^+^ T cells (93.54% ± 6.23%, *N* = 80), CD8^+^ T cells (68.33% ± 9.64%, *N* = 80), CD4^+^ T cells (28.27% ± 5.79%, *N* = 80), NK cells (3.52% ± 2.96%, *N* = 80), and NKT cells (24.33% ± 8.16%, *N* = 80). The proportion of PD-1 was expressed as mean ± SD of 20.34% ± 8.25% of infused TILs, primarily on the CD8^+^ T cells (17.25% ± 6.28%, *N* = 80). A subgroup of Foxp3^+^ T regulatory cells (18.96% ± 8.69%, *N* = 80) was measured from the CD3^+^CD4^+^ T cell population.

### 3.3. Cytotoxicity and Cytokines Production by TILs

The antitumor effects of TILs were evaluated by measuring the cytotoxicity of TILs against Hela target cell line. TILs were cocultured with Hela cells for 72 h at effector to target (E/T) cell ratios of 40 : 1, 20 : 1, and 10 : 1. Median cytotoxicity levels of TILs were 80.78% ± 5.68%, 55.34% ± 3.76%, and 38.49% ± 3.12% (Supplementary Figure 2A). TILs kill cancer cells by releasing cytokines, such as IFN-*γ*, IL-2, and TNF-*α* [26]. Flow cytometry analysis showed that cultured TILs enhanced the production of IFN-*γ*, IL-2, and TNF-*α* compared with fresh TILs (Supplementary Figure 2B and C). Taken together, these data showed that cultured TILs had the viability to kill tumor cells.

### 3.4. Treatment-Related Toxicities

The most common AEs related to the combination therapy of TILs and anti-PD-1 were fever, fatigue, rash, and anorexia (Table 2). In general, treatment-associated AEs occurred in 73 patients (91.25%), in which grade 1 or 2 AEs were observed in 69 (94.52%) of the 73 patients. Grade 3 or 4 treatment-associated AEs were observed in four patients (5.00%). One patient exhibited a grade 4 fever during treatment, but the latter underwent objective antitumor regression (complete response, CR) after 3 months of combined TILs and nivolumab therapy. One patient who developed grade 3 fever underwent CR after 3 months of the combined therapy. Grade 3 fever was also observed in the other two patients who underwent partial recovery (PR) after 6 cycles of the combined therapy. Notably, fever was the most frequently observed AE, which occurred in 50 (62.5%) patients. However, the body temperature for nearly all cases of fever did not exceed 38°C and spontaneously resolved within 12 hours. The patients with grade 3 and 4 fever were treated with nonsteroidal anti-inflammatory drugs, which resolved within 48 hours. No patient exhibited other treatment-associated serious AEs such as infectiousness, vitiligo, nausea, or vomiting.

### 3.5. Treatment Efficacy

**Figure 1:**
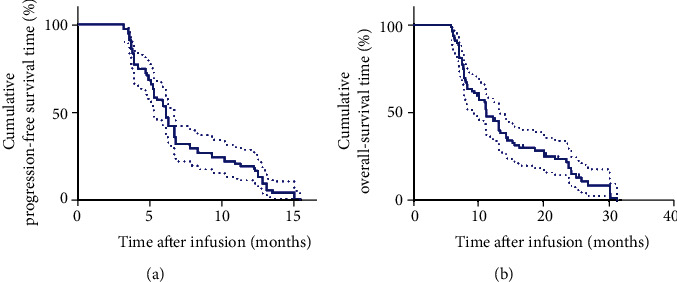
Kaplan–Meier curves for PFS and OS of metastatic cervical cancer. (a) Patients' PFS curve. (b) Patients' OS curve.

**Figure 2:**
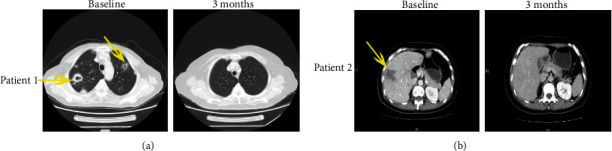
Patients exhibiting a CR of multiple lung metastases and liver metastases after TILs plus anti-PD1 therapy. (a) The patient achieved a CR of multiple lung metastases (the yellow arrows) after 3 months of immunotherapy. (b) The patient achieved a CR of multiple liver metastases (the yellow arrows) after 4 months of immunotherapy.

The ORR was observed in 20 (25.0%) out of 80 patients, in which 4 underwent CR whereas 16, exhibited PR. In addition to the response level, DCR was observed in 50 (62.5%) patients. At the end of follow-up in July 2020, all cancers had progressed significantly in all patients, and 70 patients had died of the disease. The medium time for PFS and OS was 6.1 and 11.3 months, respectively (Figures 1(a) and 1(b)). Notably, the patients who underwent CR were 35 and 50 years old, presenting with lung and liver metastases, respectively. After 12 weeks of the combined therapy, the multiple lung (Figure 2(a)) and liver metastases (Figure 2(b)) disappeared. The PFS was 15.4 months for the first patient, and 10.9 months for the second. The two patients are still alive. The median time for PFS and OS for the 20 patients with CR or PR was 12.8 and 25.8 months, respectively. Of note is that 7 of the 20 patients who underwent PR are still alive and undergoing the last follow-up.

### 3.6. The ORR and Non-ORR of the Study Cohort

**Figure 3:**
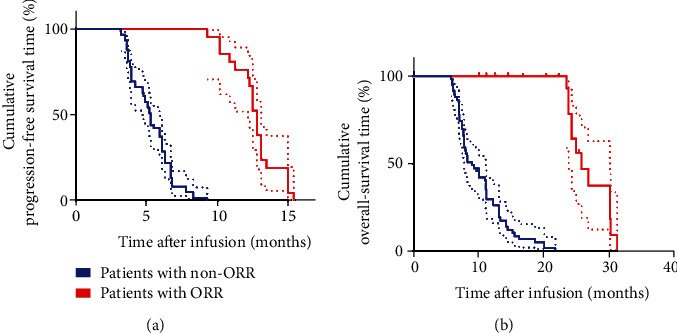
Kaplan–Meier curves for PFS and OS of patients with ORR (CR+PR), *N* = 20 and non-ORR, *N* = 60. (a) The PFS curve of patients with ORR and non-ORR. (b) The OS curve of patients with ORR and non-ORR.

The mPFS and mOS of the patients who displayed ORR (*N* = 20) and patients that did not show ORR (*N* = 60) were 12.8 months vs. 5.3 months (*P* < 0.0001) and 25.8 months vs. 9.1 months (*P* < 0.0001), respectively (Figures 3(a) and 3(b)). Notably, patients who exhibited ORR were more likely to show better response to TILs and anti-PD1 combination therapy. Thus, we further explored potential biomarkers that could predict efficacy of this therapy based on the characteristics of patients who exhibited ORR. The first analysis was performed for age, ECOG PS, size of primary tumor, pathological type, type of first-line chemotherapy (with bevacizumab or not), and location of metastatic tumors. Surprisingly, there was no significant difference among these factors between the two groups of patients (ORR and non-ORR) (Table 3). Interestingly, significant differences in the number of infusion TIL; percentage of CD8^+^ TIL, CD8^+^PD1^+^ TIL, and CD4^+^FoxP3^+^ TIL; status of HPV infection; and development of fever after immunotherapy were reported between the two groups of patients (Table 3). The number of infused TIL and percentage of CD8^+^ TIL in patients who underwent ORR versus those who did not were 50 × 10^9^ ± 5.2 × 10^9^ vs. 28 × 10^9^ ± 2.3 × 10^9^ (*P* < 0.0001) and 76.8% ± 3.5% vs. 51.3% ± 3.2% (*P* < 0.0001), respectively. Conversely, percentage of infused CD8^+^PD1^+^ TIL and CD4^+^FoxP3^+^ TIL in patients who exhibited ORR versus patients who did not were 6.2% ± 1.4% vs. 26.3% ± 2.9% (*P* < 0.0001) and 10.3% ± 1.9% vs. 25.4% ± 5.3% (*P* < 0.0001), respectively. Additionally, many patients in the ORR group developed fever and tested positive for HPV (100% vs. 50% and 100% vs. 80%), respectively. Based on these findings, higher infusion of TIL cells and the percentage of CD8^+^ TIL, less infusion of CD8^+^PD1^+^ TIL and CD4^+^FoxP3^+^ TIL, and infection with HPV and fever are potential factors predicting the response to TILs and anti-PD1 combination therapy.

### 3.7. Prognostic Factors Associated with TIL and Anti-PD1 Combination Therapy

**Figure 4:**
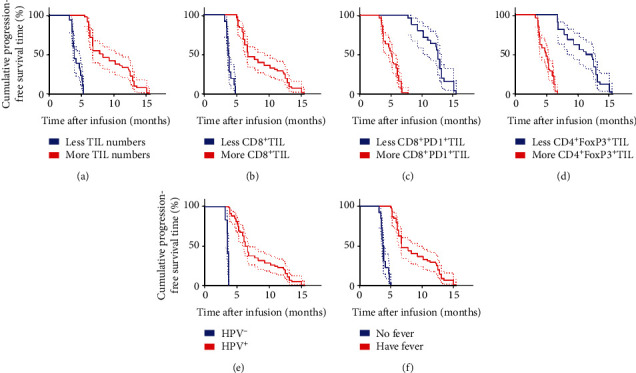
Univariate analyses of more infusion (TIL numbers and CD8^+^TIL percentage), less infusion (CD8^+^PD1^+^ TIL percentage and CD4^+^FoxP3^+^TIL percentage), and HPV status and having fever or not after immunotherapy based on PFS. PFS curve for: (a) patients with more TIL numbers (≥50 × 10^9^, red line) and less TIL numbers (<50 × 10^9^, blue line); (b) patients with more CD8^+^ TIL (≥60%, red line) and less CD8^+^ TIL (<60%, blue line); (c) patients with more CD8^+^PD1^+^ TIL (≥10%, red line) and less CD8^+^PD1^+^ TIL (<10%, blue line); (d) patients with more CD4^+^FoxP3^+^ TIL (≥20%, red line) and less CD4^+^FoxP3^+^ TIL (<20%, blue line); (e) patients with HPV^+^ (red line) and HPV^−^ (blue line); and (f) patients having fever (red line) and no fever (blue line) after immunotherapy.

**Figure 5:**
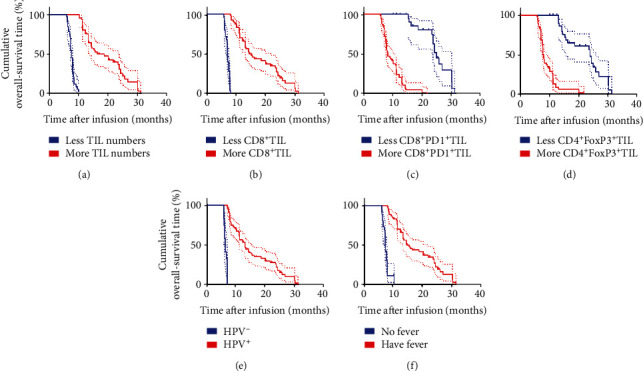
Univariate analyses of more infusion (TIL numbers and CD8^+^ TIL percentage), less infusion (CD8^+^PD1^+^ TIL percentage and CD4^+^FoxP3^+^ TIL percentage), and HPV status and having fever or not after immunotherapy based on OS. OS curve for: (a) patients with more TIL numbers (≥50 × 10^9^, red line) and less TIL numbers (<50 × 10^9^, blue line); (b) patients with more CD8^+^ TIL (≥60%, red line) and less CD8^+^ TIL (<60%, blue line); (c) patients with more CD8^+^PD1^+^ TIL (≥10%, red line) and less CD8^+^PD1^+^ TIL (<10%, blue line); (d) patients with more CD4^+^FoxP3^+^ TIL (≥20%, red line) and less CD4^+^FoxP3^+^ TIL (<20%, blue line); (e) patients with HPV^+^ (red line) and HPV^−^ (blue line); and (f) patients having fever (red line) and no fever (blue line) after immunotherapy.

There was higher infusion of TIL, higher percentage of CD8^+^TIL but lower infusion of CD8^+^PD1^+^ and CD4^+^FoxP3^+^ TIL in patients who displayed ORR. Additionally, patients with ORR were all positive for HPV and developed fever after immunotherapy. Based on this, we assessed potential prognostic factors that may predict clinical response to the combined therapy. Moreover, Kaplan–Meier analysis revealed that no significant difference in medium PFS and OS for different age groups, ECOG PS, size of primary tumor, pathological type, first-line chemotherapy (with bevacizumab or not), and location of metastatic tumors (Table 4). In contrast, univariate analyses revealed that higher infusion of TIL and CD8^+^ TIL percentage, lower infusion of CD8^+^PD1^+^ and CD4^+^FoxP3^+^ TIL, and infection with HPV and development of fever were significantly associated with higher medium PFS (8.3 months vs. 3.9 months, *P* < 0.0001; 6.8 months vs. 3.8 months, *P* < 0.0001; 12.5 months vs. 5.2 months, *P* < 0.0001; 11.75 months vs. 4.9 months, *P* < 0.0001; 6.5 months vs. 3.6 months, *P* < 0.0001; and 6.8 months vs. 3.85 months, *P* < 0.0001) (Figures 4(a)–4(f)), as well as medium OS (18.7 months vs. 7.6 months, *P* < 0.0001; 15.3 months vs. 7.1 months, *P* < 0.0001; 25.0 months vs. 8.4 months, *P* < 0.0001; 23.8 months vs. 8.0 months, *P* < 0.0001; 13.2 months vs. 6.45 months, *P* < 0.0001; and 15.3 months vs. 7.15 months, *P* < 0.0001) (Figures 5(a)–5(f)). Similar findings were obtained in the multivariate Cox proportional hazard model (*P* < 0.0001) for both median PFS (Table 5) and OS (Table 6). In conclusion, higher infusion of TIL and CD8^+^ TIL, lower infusion of CD8^+^PD1^+^ TIL and CD4^+^FoxP3^+^ TIL, and infection with HPV and occurrence of fever are potential factors for predicting the clinical response to combination therapy of TILs and anti-PD1.

## 4. Discussion

Although many clinical studies have demonstrated long-term survival benefits of immunotherapy in various cancer entities, little is known whether this therapy is effective against cervical cancers. In the United States, pembrolizumab, an antibody against 1 PD-1, is approved for advanced endometrial cancers with high levels of microsatellite instability (MSI-high) and for recurrent or progressive metastatic cervical cancer positive for programmed death-ligand 1 (PD-L1). This suggests that only a specific group of patients benefit from this immunotherapy [27]. Patients with metastatic human papillomavirus (HPV)-associated carcinomas put on TILs therapy have shown modest response to the treatment [19]. However, effective immunotherapies for the treatment of metastatic cervical cancers accompanied with low MSI expression and negative for PDL1 are limited. Here, we explored the efficacy of adoptive transfer of TILs and anti-PD1 antibody in patients with metastatic cervical cancer showing low MSI expression and negative for PDL1. Interestingly, we found that this combination therapy registered promising antitumor effects and a satisfactory objective response, with clinical tumor regression observed in 20 out of the 80 patients (25.0%). These findings suggest that a combination therapy of TILs and anti-PD1 may potentially modulate the growth of metastatic cervical cancers in patients with low MSI expression and negative for PDL1.

This research was motivated by the lack of effective treatment options for metastatic cervical cancers, particularly in patients who fail to respond to first-line therapy. Out of the 80 patients, 20 showed objective response, in which 4 underwent CR, whereas 16 exhibited PR. The medium PFS and OS were 6.1 and 11.3 months, respectively. Previous studies (KEYNOTE-028 and KEYNOTE-158) have reported considerable benefits of anti-PD1 therapy in controlling persistent and advanced cervical cancer [28, 29]. In these studies, however, patients with lacking PD-L1 expression did not respond to immunotherapy. Despite being a single-center, single-arm retrospective clinical study, our findings suggest that a combination therapy of TILs and anti-PD1 may be effective against metastatic cervical cancers resistant to chemotherapy, and in patients with low MSI expression and PDL1-negative.

The effects of anti-PD1 therapy are mediated by TILs in the tumor microenvironment; therefore, combining anti-PD1 with TIL therapy may confer more effective antitumor effects on metastatic cervical cancer. Previous studies have shown that IFN-*γ*, an inflammatory cytokine, produced by TILs induces expression of PD-L1 in tumor cells [30]. Although, we did explore the mechanism underlying ORR in the 20 patients who showed positive response to the therapy; we speculate that TILs penetrate the tumor microenvironment and secrete IFN-*γ*, which induces expression of PD-L1 in the tumor cells. Cancer cells expressing this ligand are thus recognized, bound by PD1 antibodies, and eventually killed by Ab-mediated processes. Further studies should be conducted to explore the effect of adoptive transfer of TILs in ablating tumors when combined with anti-PD1 therapy.

The most common treatment-related adverse events of any grade were fever, fatigue, rash, and anorexia. There were few AEs of grade 3 or 4, especially when considered in the context of the cytotoxic chemotherapy agents that patients in this population had previously received. Four patients who exhibited grade 3 or 4 fever were effectively treated with nonsteroidal anti-inflammatory drugs and resolved within 48 hours. Interestingly, two of the four patients underwent CR. Univariate and multivariate analyses showed that patients who developed fever after immunotherapy exhibit longer PFS and higher OS. In one study, it was found that fever after anti-PD1 therapy may be an early predictor for better response to anti-PD1 treatment; this is in agreement with our findings. However, future studies should focus on exploring the association between fever and immunotherapy.

Persistent HPV infection influences the pathogenesis and prognosis of cervical cancer. Whereas HPV-associated cancers constitutively express HPV E6 and E7 oncoproteins; immunologically foreign viral proteins that are attractive targets for immunotherapy [31], targeting HPV oncoproteins with antigen-specific immunotherapy using therapeutic vaccines has so far been ineffective for metastatic disease [19]. One study suggested that the association between HPV status with the efficacy of PD-1/PD-L1 inhibitors against cervical cancer is not clear due to the paucity of available data [16]. Nonetheless, cultured TILs have been shown to mediate the regression of HPV-associated epithelial cancers including cervical cancer [19, 20]. Moreover, the reactivity of HPV with the infused T cells together with repopulation of peripheral blood with HPV-reactive T cells also correlated with positive clinical response [19]. In this study, univariate and multivariate analyses showed that patients not infected with HPV exhibited better PFS and OS. Based on this finding, HPV status may predict the prognosis of patients following immunotherapy.

Consistent with previous findings [23, 24], univariate and multivariate analyses indicated that patients with higher infusion of TILs and CD8^+^ TILs exhibited better PFS and OS. Conversely, higher infusion of CD8^+^PD1^+^ TIL and CD4^+^FoxP3^+^ TIL resulted in poor PFS and OS. Expression of PD1 by TILs is thought to be one of the factors that weaken antitumor immune response [32]. Consistently, individuals with higher infusion of CD8^+^PD1^+^ TIL exhibited poor PFS and low OS. One recent study reported that blocking the PD-1 pathway significantly increased antitumor effects of adoptive immunotherapy performed using T cells with chimeric antigen receptor (CAR) [33]. In this study, a subpopulation of PD-1^+^ T lymphocytes was observed in the cultured TILs, suggesting that a PD-1 blockade may significantly increase the cytotoxicity of TILs. Regulatory T cells (Tregs) have been shown to suppress T cell-mediated host immune response against self- and nonself-antigens [34–36]. In fact, studies have described a negative relationship between peripheral CD4^+^FoxP3^+^ regulatory T cell levels and clinical response to adoptive immunotherapy of human cancer [37], suggesting that Tregs mediate the inhibitory effects of adoptive immunotherapy on human tumors. In this study, we found that higher infusion of CD4^+^FoxP3^+^ TIL resulted in poor PFS and OS, suggesting that eliminating the suppressive T-regulatory lymphocytes can enhance antitumor efficacy.

This study was carried out in a single-center, with single-arm and retrospective clinical design. However, we show that a combination therapy of TILs and anti-PD1 may potentially inhibit the growth of metastatic cervical cancers in patients with low MSI expression and negative for PDL1. However, whether TILs should be administered in combination with anti-PD-1 or as a single treatment option in patients with metastatic cervical cancer is still unclear. Further studies should be conducted to clarify this. Moreover, the reliability of using cultured TILs as biomarkers for predicting clinical response to the combination immunotherapy should be further investigated.

## 5. Conclusion

In conclusion, our results show that a combination therapy of TILs with anti-PD1 presents an effective approach for controlling metastatic cervical cancer in patients with low MSI-l and PDL1 negative. However, further studies are needed to validate these findings and describe the specific mechanisms underlying the use of TILs anti-PD1 therapy.

## Figures and Tables

**Table 1:** Patient characteristics (*N* = 80).

Characteristic	No. of patients	%
Age (years)		
≥45	45	56.25
<45	35	43.75
Pathological type		
Squamous cell	65	81.25
Adenocarcinoma	15	18.75
ECOG PS		
0	50	62.50
1	30	37.50
HPV status		
Positive	68	85.00
Negative	12	15.00
Size of primary tumor(cm)		
≥5	47	58.75
<5	33	41.25
First-line chemotherapy with bevacizumab		
Yes	20	25.00
No	60	75.00
Location of metastatic tumors		
Lung	55	68.75
Liver	30	37.50
Bone	20	25.00
Lymph node	40	50.00
Peritoneum	35	43.75

**Table 2:** Treatment-related adverse events in patients in response to therapy (*N* = 80).

Sides effects	No. (%) patients associated with adverse events	
	Grade 1or 2	Grade 3 or 4
Fever	50 (62.50)	4 (5.00)
Fatigue	18 (22.50)	0
Rash	16 (20.00)	0
Anorexia	12 (15.00)	0
Leukopenia	5 (6.25)	0
Anemia	4 (5.00)	0
Vitiligo	0	0
Nausea	0	0
Vomiting	0	0
Total incidence	69 (86.25)	4 (5.00)

**Table 3:** Characteristics of patients with ORR (*N* = 20) and non-ORR (*N* = 60).

Characteristic	No. of ORR	No. of non-ORR	*P* value
Age (years)			
≥45	11	34	
<45	9	26	0.896
Pathological type			
Squamous cell	16	49	
Adenocarcinoma	4	11	0.869
ECOG PS			
0	13	37	
1	7	23	0.790
Size of primary tumor (cm)			
≥5	9	38	
<5	11	22	0.149
First-line chemotherapy with bevacizumab			
Yes	8	12	
No	12	48	0.074
Location of metastatic tumors			
Lung	11	45	
Others	9	15	0.091
Infusion of TIL numbers			
≥50 × 10^9^	17	10	
<50 × 10^9^	3	50	0.000
Infusion of CD8^+^ TIL percentage			
≥60%	18	11	
<60%	2	49	0.000
Infusion of CD8^+^PD1^+^ TIL percentage			
≥10%	3	35	
<10%	17	25	0.001
Infusion of CD4^+^FoxP3^+^ TIL percentage			
≥20%	4	33	
<20%	16	27	0.007
HPV status			
Positive	20	48	
Negative	0	12	0.030
Fever			
Yes	20	34	
No	0	26	0.000

**Table 4:** Univariate analysis of factors related to mPFS and mOS of patients in this study (N=80).

Characteristics	mPFS (months)	*P* value	mOS (months)	*P* value
Age (years)				
≥45	5.50		10.2	
<45	6.80	0.613	13.4	0.395
Pathological type				
Squamous cell	6.10		11.3	
Adenocarcinoma	3.90	0.639	7.60	0.791
ECOG PS				
0	7.30		13.4	
1	5.90	0.562	11.2	0.823
Size of primary tumor (cm)				
≥5	6.70		13.2	
<5	6.10	0.115	11.2	0.895
First-line chemotherapy with bevacizumab				
Yes	6.30		11.3	
No	4.45	0.400	8.00	0.157
Location of metastatic tumors				
Lung	8.30		15.3	
Others	6.10	0.931	11.3	0.130

**Table 5:** Multivariate analysis (mPFS).

Parameters	Hazard ratio	95% confidence interval	*P* value
Infusion of CD8^+^ TIL numbers (≥50 × 10^9^ vs. <50 × 10^9^)	5.823	(2.871, 11.81)	<0.0001
Infusion of CD8^+^ TIL percentage (≥60% vs. <60%)	7.05	(2.813, 17.67)	<0.0001
Infusion of CD8^+^PD1^+^ TIL percentage (≥10% vs. <10%)	4.285	(2.697, 6.807)	<0.0001
Infusion of CD4^+^FoxP3^+^ TIL percentage (≥20% vs. <20%)	4.551	(2.74, 7.561)	<0.0001
HPV status (positive vs. negative)	11.84	(1.915, 73.16)	<0.0001
Fever (yes vs. no)	6.857	(2.83, 16.62)	<0.0001

**Table 6:** Multivariate analysis (mOS).

Parameters	Hazard ratio	95% confidence interval	*P* value
Infusion of CD8^+^ TIL numbers (≥50 × 10^9^ vs. <50 × 10^9^)	6.169	(3.001, 12.68)	<0.0001
Infusion of CD8^+^ TIL percentage (≥60% vs. <60%)	7.569	(2.992, 19.15)	<0.0001
Infusion of CD8^+^PD1^+^ TIL percentage (≥10% vs. <10%)	5.321	(3.29, 8.606)	<0.0001
Infusion of CD4^+^FoxP3^+^ TIL percentage (≥20% vs. <20%)	4.916	(2.939, 8.222)	<0.0001
HPV status (positive vs. negative)	12.74	(2.967, 82.5)	<0.0001
Fever (yes vs. no)	6.314	(2.674, 14.91)	<0.0001

## Data Availability

All data generated in the study are included in the present article.

## Supplementary Materials

Supplementary 1 Supplementary Figure 1: representative flow cytometry of CD3^+^, CD3^+^CD4^+^, CD3^+^CD8^+^, CD3^+^CD56^+^, CD3^−^CD56^+^, CD3^+^CD8^+^PD1^+^, CD4^+^FoxP3^+^ percentage of TILs.

Supplementary 2 Supplementary Figure 2: cytotoxicity and cytokines production by TILs. (A) The cytotoxicity of TILs against Hela target cell line; (B) representative flow cytometry of IFN-*γ*, IL-2, and TNF-*α* expression on CD3^+^ fresh TILs from biopsy samples and cultured TILs. (C) Quantitative analysis of IFN-*γ*, IL-2, and TNF-*α* expression on CD3^+^ fresh TILs from biopsy samples and cultured TILs.
