# Supplementary figures and images for: TILs and Anti-PD1 Therapy: An Alternative Combination Therapy for PDL1 Negative Metastatic Cervical Cancer
Source: J Immunol Res. 2020 Sep 7;2020:8345235. doi: 10.1155/2020/8345235 (PMC7492938; doi:10.1155/2020/8345235)

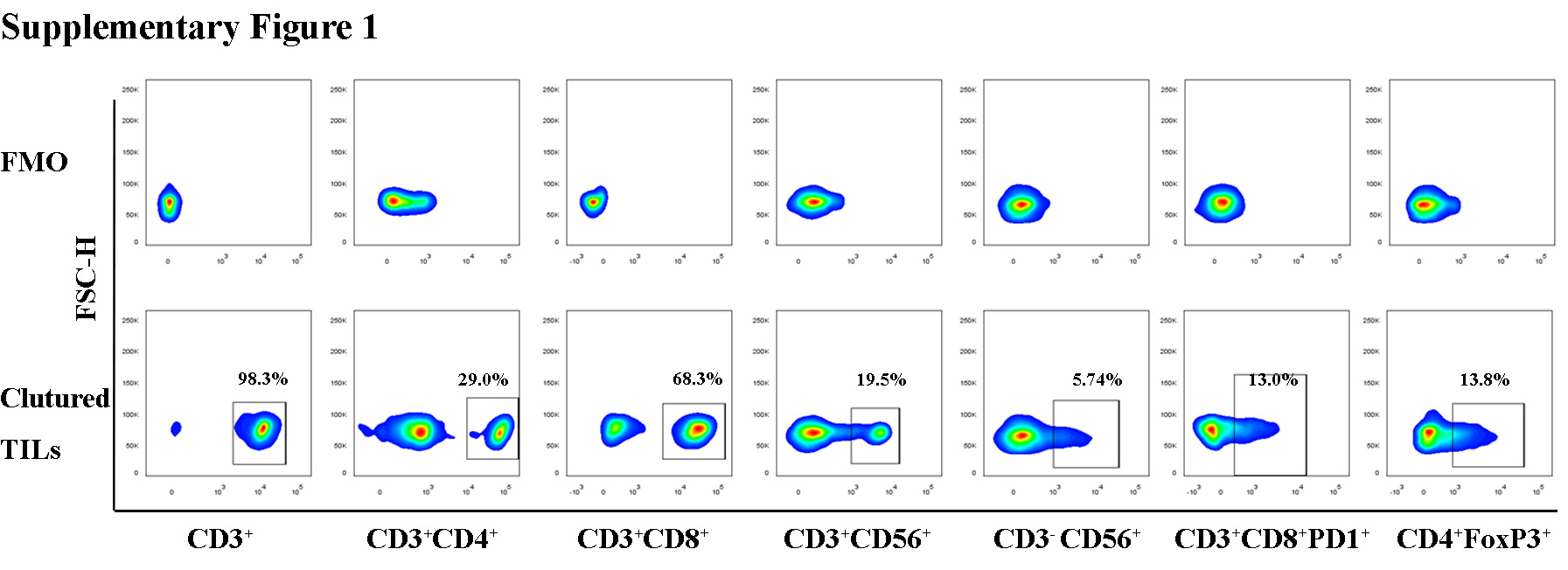

Supplement: Supplementary 1 — Supplementary Figure 1: representative flow cytometry of CD3+, CD3+CD4+, CD3+CD8+, CD3+CD56+, CD3−CD56+, CD3+CD8+PD1+, CD4+FoxP3+ percentage of TILs. [file 8345235.f1.jpg]

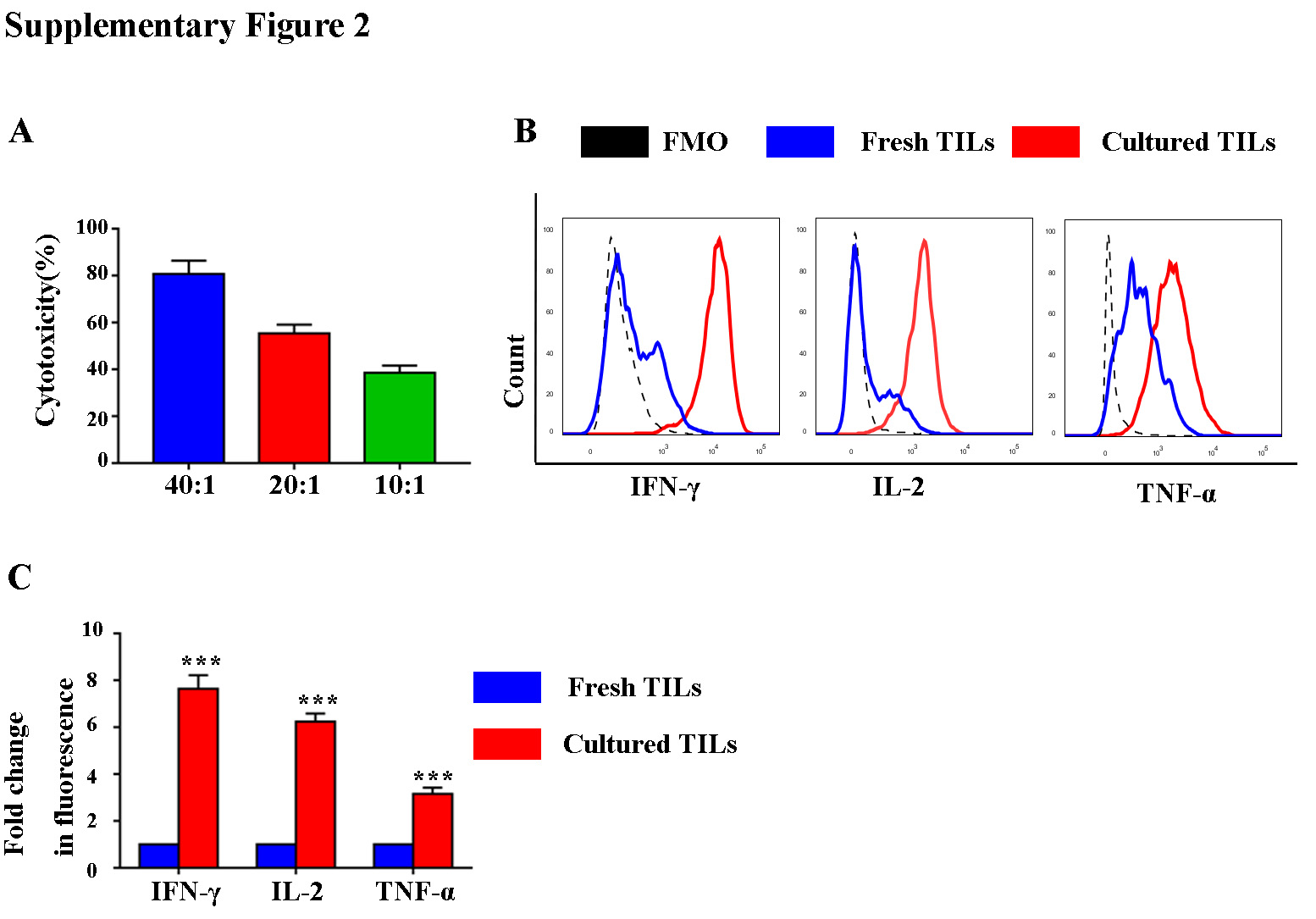

Supplement: Supplementary 2 — Supplementary Figure 2: cytotoxicity and cytokines production by TILs. (A) The cytotoxicity of TILs against Hela target cell line; (B) representative flow cytometry of IFN-γ, IL-2, and TNF-α expression on CD3+ fresh TILs from biopsy samples and cultured TILs. (C) Quantitative analysis of IFN-γ, IL-2, and TNF-α expression on CD3+ fresh TILs from biopsy samples and cultured TILs. [file 8345235.f2.jpg]
